# Thermoelectric heat exchange and growth regulation in a continuous yeast culture

**DOI:** 10.1002/mbo3.648

**Published:** 2018-05-24

**Authors:** Adriel Latorre‐Pérez, Cristina Vilanova, José J. Alcaina, Manuel Porcar

**Affiliations:** ^1^ Darwin Bioprospecting Excellence SL Paterna Spain; ^2^ Biotechnology and Synthetic Biology Laboratory I2SysBio (Institute for Integrative Systems Biology) University of Valencia‐CSIC Paterna Spain

**Keywords:** continuous culture, heat exchange, Peltier–Seebeck effect, power production, temperature regulation

## Abstract

We have designed a thermoelectric heat exchanger (TEHE) for microbial fermentations that is able to produce electric power from a microbial continuous culture using the intrinsic heat generated by microbial growth. While the TEHE was connected, the system proved able to stably self‐maintain both the temperature and the optical density of the culture. This paves the way toward a more sustainable operation of microbial fermentations, in which energy could be saved by converting part of the metabolic heat into usable electric power.

## 1 INTRODUCTION

A range of parameters such as temperature, pH, or substrate concentration need to be stable in order to sustain a suitable microbial growth and/or a stable biosynthesis of a bioproduct (Walker, 2000). Temperature strongly affects a range of fundamental cellular processes (Goldberg, 2003; Haas, 2010), and thus keeping a microbial culture in a suitable range of temperatures is of high importance in terms of strain performance (Amillastre, Aceves‐Lara, Uribelarrea, Alfenore, & Guillouet, 2012). Large‐scale growth of most microorganisms is accompanied by the production of heat (Brettel, Lamprecht, & Schaarschmidt, 1981), which, when large culture volumes are set, often results in an undesirable increase in the temperature of the batch culture that has to be alleviated through refrigeration (von Stockar & van der Wielen, 1997; Türker, 2004).

In a previous work, we described the first microbial thermoelectric cell (MTC), a system designed for batch cultures that allows the partial conversion of microbial metabolic heat into electricity. MTC is based on the Seebeck effect, a thermoelectric property that allows direct conversion of temperature differences to electricity voltage. Taking into account that microbial growth is mainly exothermic, theoretically it is possible to produce an electrical current with the generated metabolic heat by using a thermoelectric cell (Rodríguez‐Barreiro, Abendroth, Vilanova, Moya, & Porcar, 2013). Nevertheless, a range of industrial fermentations are carried out in continuous culture, where stable cellular densities can be maintained during long periods thanks to the supply of fresh medium, which is introduced at a rate that is equal to the volume of product that is removed from the fermenter. In this work, we aimed at designing, constructing, and characterizing a continuous culture system in which temperature is automatically controlled and electric power is constantly obtained during all the fermentation process. To do that, we envisaged, constructed, and set in place a thermoelectric heat exchanger (hereafter called TEHE), a device also based on the Seebeck effect, which facilitates a fine control of temperature and fresh medium input—and thus microbial growth—while electric power is produced.

## 2 MATERIALS AND METHODS

### 2.1 Experimental set‐up

**Figure 1:**
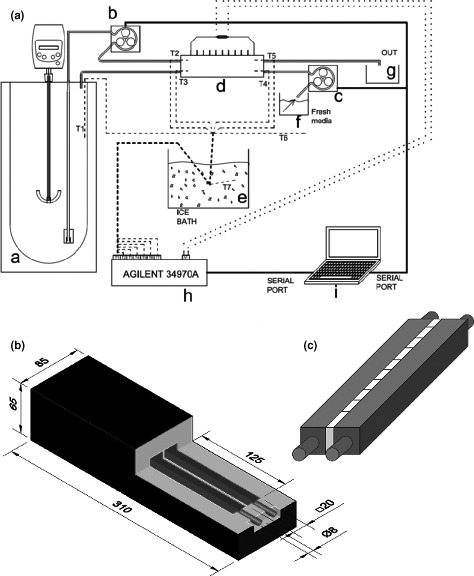
(A) Schematic representation of the continuous culture system. (a) Thermally insulated fermenter; (b,c) peristaltic pumps; (d) Thermoelectric heat exchanger (TEHE); (e) ice bath for the compensation of temperature measurements; (f) refrigerated fresh medium tank; (g) wasted medium tank; (h) data logger; and (i) PC with a software (Rodríguez‐Barreiro et al., 2013) for data recording and automatic control of the peristaltic pumps. All temperature measurements were performed with T‐type thermocouples. The points at which each temperature was measured are represented as T(1–7). (B) Tridimensional representation of the TEHE constructed in this work. Sizes given in mm. (C) Zoom‐in detail of the aluminum pipes inserted in the TEHE, with the thermoelectric generator cells in the interphase between both pipes

A medium‐scale continuous culture of budding yeast *Saccharomyces cerevisiae* strain D170 (kindly provided by Prof. Emilia Matallana, IATA, Valencia, Spain) in YPD medium supplemented with 18% sucrose was set up in the laboratory as schematically represented in Figure 1A. The TEHE consisted of two aluminum pipes of squared section and a serial connection of 10 Peltier coolers (MCPE‐071‐10‐13, Multicomp) placed in direct contact with the pipes. The whole device was thermally insulated with expanded polystyrene and polyurethane foam spray (Silicex Fischer, Fisher Ibérica, Tarragona, Spain) (Figure 1B). The TEHE was coupled to a thermally isolated 40 L Dewar flask (Scharlab, Barcelona, Spain) combined with a MM‐1000 overhead anchor stirrer (Labnet International, Edison, NJ), and two peristaltic pumps (Lambda Laboratory Instruments, Baar, Switzerland), which were programmed to control the flow of fresh and wasted medium.

### 2.2 Data acquisition, monitoring and recording

The whole system was connected to a PC in order to record temperature values as well as output electrical current. Temperature measurements were performed by thin T‐type thermocouples inserted into the different parts of the system and connected to a PC through a data logger. As in previous studies (Rodríguez‐Barreiro et al., 2013), the connections between the thermocouples and the data logger were performed on an ice‐water mixture, which was used only as a thermal reference, to take into account the unwanted electric voltage background due to the junction of dissimilar metals in the thermocouple data logger connection. Temperature and electrical current records were taken every 6 min throughout the experiment. Both feed and effluent flows were automatically modulated during all the experiment with the LabVIEW control software.

### 2.3 Mathematical modeling

The global thermal resistance (*R*g) (K/W) and the whole heat capacity (*m·c*p) (J/K) of the fermenter were estimated from Equation (1):

(1) $$m\cdot c_{p}\cdot \frac{dT\text{ce}}{dt}\;=\;Q\cdot \rho \cdot c_{p}\cdot \left(T\mathrm{fs}-T\mathrm{ce}\right)\;+\;Pc\;-\;\frac{T\mathrm{ce}-T\mathrm{env}}{R_{g}}$$

where *m*,* c*p, *ρ,* and *T*ce are broth mass (kg), specific heat (J/kg·K), density (kg/m^3^), and temperature of the hot inlet flow (K), respectively, whereas *Q* is the flow rate (m^3^/s) and *P*c is the metabolic heat produced by yeasts (W). *T*fs (K) and *T*env (K) correspond to the fresh medium entering the fermenter and room temperatures, respectively.

Equations (2) and (3), describing a logarithmic mean temperature difference (LMTD) model of a heat exchanger (Mizutani, Pessoa, Queiroz, Hauan, & Grossmann, 2003), were used to mathematically characterize the TEHE:

(2) $$\mathrm{LMTD}\;=\;\Delta T_{\ln}\;=\;\frac{\left(T\mathrm{ce}\;-\;T\mathrm{fs}\right)-\left(T\mathrm{cs}-T\mathrm{fe}\right)}{\ln\left(\frac{T\mathrm{ce}\;-\;T\mathrm{fs}}{T\mathrm{cs}\;-\;T\mathrm{fe}}\right)}$$

(3) q=U·A·LMTD

*T*ce and *T*fe being the temperature of the hot and cold inlet flows, respectively; and *T*cs and *T*fs, the temperature of the hot and cold outlet flows, respectively. The *q* term (W) is the heat flow between the hot and the cold pipe, and it depends on the temperature of the fluids entering the exchanger. *U* is the global heat transmission coefficient (W/m^3^), and *A* (m^3^) is the heat exchange surface.

In order to obtain an estimation of the *U·A* constant, *q* was first calculated from Equation (4) in an experiment where two water flows at known temperatures were inserted in the TEHE.

(4) $$q\;=\;\overset{´}{m}\cdot c_{p}\cdot \left(T\mathrm{ce}-T\mathrm{cs}\right)\;=\;\overset{´}{m}\cdot c_{p}\cdot \left(T\mathrm{fs}-T\mathrm{fe}\right)$$

where *ḿ* is the inlet mass flow rate (mass of water entering the TEHE per unit of time), and *c*p is the specific heat of water (J/kg·K).

## 3 RESULTS AND DISCUSSION

**Figure 2:**
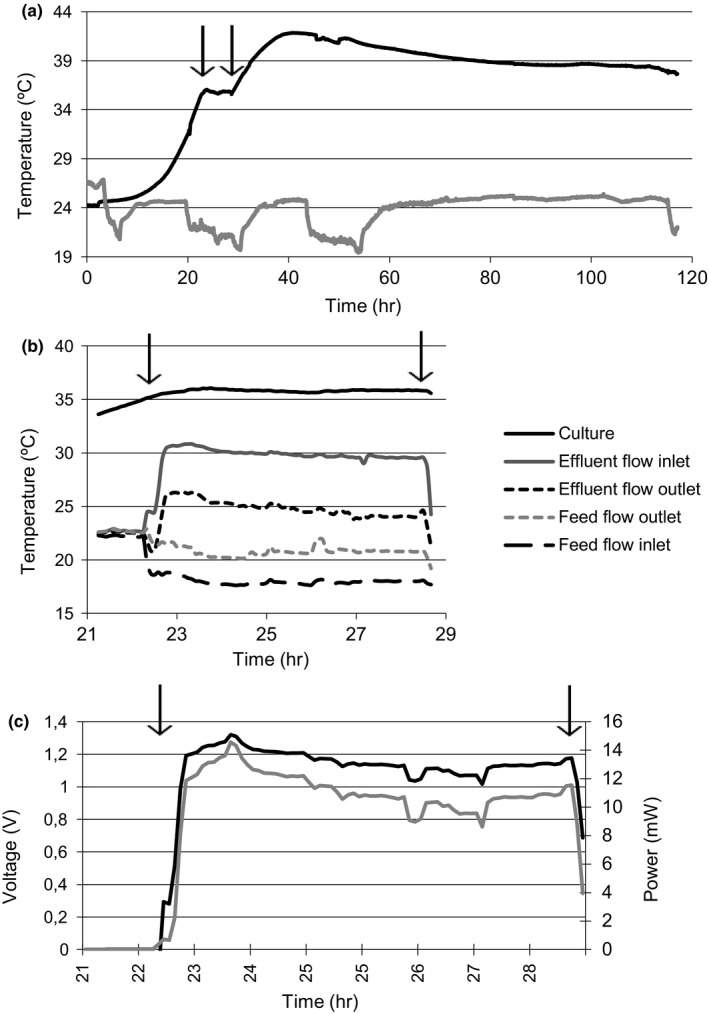
(a) Evolution of broth (black line) and room temperatures (gray line) in a typical experiment. Arrows indicate the period of time when the thermoelectric heat exchanger (TEHE) was connected. (b) Changes in the temperature of inlet and outlet flows of the TEHE. (c) Voltage (black line) and power (gray line) production in the TEHE

The output of a typical experiment carried out in the continuous culture system set as described above is shown in Figure 2. The broth (35 L) was inoculated with 700 ml (1:50) of an overnight yeast culture, and cultivated in the thermally isolated flask under shaking (180 rpm). Broth temperature rose in an exponential fashion and reached 35°C after 24 hr, (Figure 2a). At this point, culture temperature was kept constant by means of introducing fresh, cool medium in the fermenter (feed flow) at the same rate that wasted (warm) medium was extracted (effluent flow), in such a way that the volume of the culture did not change during the experiment. The heat flowing from the warm side to the cold side of the TEHE heated the fresh medium (from 18 up to 22°C, approximately) prior to its entrance to the fermenter (Figure 2b); and, reciprocally, cooled the warm waste medium (with a temperature at the TEHE input of around 30°C) down to around 25°C, approximately. As a result, a rather stable voltage of 1–1.3 V was recorded (Figure 2c). In order for the TEHE to produce the maximum electric power, a load resistance of 120 Ω was coupled to the terminals of the thermoelectric generators yielding 10–12 mW. When the feed and effluent flows were halted and the TEHE was not used, the temperature of the broth started rising immediately, peaked at 42°C, and then started to drop (Figure 2a).

The thermal behavior of the two main components of the system (the fermenter and the TEHE) was experimentally characterized and mathematically modeled. Following a simplified experimental set up where no fresh medium (*Q*=0) nor cells (*P*c=0) were introduced in the fermenter, an identification assay was performed to estimate *Rg* and *m·c*p, obtaining values of 5.92 K/W and 146,547 kJ/K, respectively. The *U·A* constant was also estimated as explained in the Materials and Methods section, yielding a value of 1,039 W/K.

**Figure 3:**
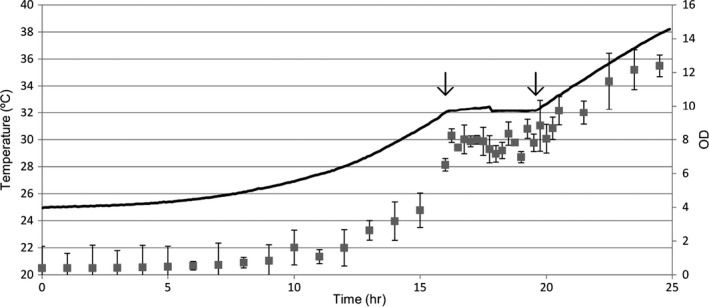
Evolution of broth temperature (black line) and optical density at 600 nm (gray squares). Arrows indicate the interval of time when the thermoelectric heat exchanger was connected. Error bars show the standard deviation of three independent measurements

The evolution of the yeast culture was studied in a typical experiment where optical density (OD) at 600 nm was periodically measured. As shown in Figure 3, yeast population and temperature during the first part of the experiment exhibited a similar pattern. When the TEHE was connected and temperature was kept constant, the OD600 of the broth was relatively stable at around eight, indicating that the number of cells present in the fermenter was maintained stable despite the large flow of broth removal (2.4 L/hr on average).

Taken together, our results prove the ability of this TEHE‐based system to regulate the broth temperature and to produce electric power by harvesting metabolic heat. In our prototype, autonomous heating of the culture was achieved and reached values (42°C) well beyond optimal temperatures for budding yeast. Lower, industrially friendly temperatures could be constantly maintained by means of an automatic equilibrium between the flow of fresh and product‐containing media through the TEHE. This resulted in the production of a significant electric power during all the process. In addition, biomass concentration proved to be constant when the temperature was controlled. This is of key importance, since industrial bioprocesses require stable temperatures in order to maintain a constant output of a given product (Park, Lee, Chang, & Chang, 1999).

The generation of electric power in heat exchangers through the Peltier–Seebeck effect has been previously reported (Bell, 2008; Riffat & Ma, 2003; Takahashi et al., 2013). However, this is, to the best of our knowledge, the first time that a heat exchanger with thermoelectric generating ability has been coupled to a bioprocess. The difference of temperatures that can be achieved between both sides of the thermoelectric generators is obviously limited by the narrow range of temperatures mesophiles can tolerate and, therefore, the electric power that can be produced is lower than that of other type of industrial processes (Weng & Huang, 2013). Albeit low, TEHE‐based power production proved to be two times more efficient compared to our previous MTC designed for batch culture: 255 μW were obtained from a 1.7 L yeast culture with the MTC, whereas TEHE produced up to 12 mW from a 35‐L culture. Thus, a 40‐fold increase in electrical power production was obtained in the TEHE compared to MTC, which was 20‐fold smaller (Rodríguez‐Barreiro et al., 2013). The metabolic heat produced by yeast growth was calculated from Equation (1), using the estimated values for *R*g and *c*_p_. According to our data, 24.36 W of metabolic heat were produced under our experimental conditions. Taking into account the power production obtained in the TEHE, we estimated that the heat conversion efficiency in our system was around 0.05%.

Our results are the first step toward thermoelectric power production coupled to industrial bioprocesses. The electric power obtained under our experimental set up was only a small percentage of the energy consumed to stir and pump the culture. However, it has to be noted that significant power production could be achieved by scaling up fermentation volumes, which might allow the design of a TEHE with an increased heat interchange surface, and more thermoelectric generators. Also, the TEHE design could be adapted to large‐scale batch fermentations, by implementing it in the physical interphase between the fermenter itself and its cooling jacket, where a significant temperature gradient takes place. This might allow an, at least, partial self‐control of the culture based on its temperature, with the TEHE contributing to power the stirrer or the peristaltic pumps that regulate broth renovation rates.

## CONFLICT OF INTEREST

The technology described in this work is subjected to industrial protection (Patent Cooperation Treaty reference number: PCT/ES2013/000212).
